# Foundational values for public health

**DOI:** 10.1186/s40985-015-0004-1

**Published:** 2015-05-29

**Authors:** Lisa M Lee, Christina Zarowsky

**Affiliations:** 1 U.S. Presidential Commission for the Study of Bioethical Issues, 1425 New York Ave., NW, Suite C-100, Washington, DC USA; 2 Centre de recherché du Centre hospitalier de l’Université de Montréal (CR-CHUM), and School of Public Health, University of Montreal, Montreal, Canada

**Keywords:** Ethics, Public health, Values

## Abstract

The development of an agreed-upon set of foundational ethical values for the field of public health is ongoing. In this paper we outline key elements of recent convergence on some basic moral precepts that drive public health. We suggest that three elements are particularly useful for anchoring public health practitioners’ reflections on public health ethics: 1) the notions of “common” and “professional” morality, 2) an understanding of the practice and content of modern public health and especially its practical, solution-focused orientation, and 3) an appreciation of the history of public health as integrally linked to evolving and contested views of the relationship between citizens, science, and the state. There is broad agreement that governments are stewards of their populations and are responsible for providing conditions that allow for its members to be healthy and productive. Given the role of policy and government in public health, the role of political philosophy likely has a substantial place as we seek a coherent system of ethical justification in our work. The aim here is not to align with one theoretical approach or another, rather, to consider the foundational values of public health practice order to identify the common moral governance of our work. Our profession’s morality—the set of norms shared by all public health professionals—is determined by what public health is and what we think it should be. As our aspirations for public health evolve, it is incumbent upon us to engage in reflective discourse to reach a new equilibrium about our moral foundation.

## Introduction

From the inception of modern public health in the 17^th^ century, it has been clear that the tools for our practice often differ from those of clinical medicine. And, while it took over three centuries to articulate, it also has become clear that the ethical tools we use to make ethically-supported decisions in public health also differ from those used in clinical medicine. Exactly what those ethical tools are has been a topic of vigorous discussion since public health ethics splintered off from clinical ethics in the mid-1980s. In early 2015, we have not yet arrived at a consensus theory, framework, or approach to modern public health ethics. We have, however, begun to see consensus on some basic moral precepts that drive public health [1].

In this paper we outline key elements of this consensus. We suggest that three elements are particularly useful for anchoring public health practitioners’ reflections on public health ethics: 1) the notions of “common” and “professional” morality, 2) an understanding of the practice and content of modern public health and especially its practical, solution-focused orientation, and 3) an appreciation of the history of public health as integrally linked to evolving and contested views of the relationship between citizens, science, and the state.

## Review

### Practical principles and foundational norms

Over the past two decades, many scholars have suggested approaches to motivate and guide the ethics of public health. Suggestions have been made from every camp, including theory-based (public health is rooted in consequentialism, based on utilitarian ethics, searching for the most good for the largest number of people); principle-based (public health interventions ought to be guided by certain agreed-upon principles such as the principles of least infringement and proportionality); case-based (examination of particulars of specific cases of public health ethical dilemmas to guide decisions in similar circumstances) and everything in between [1]. At the root of these various approaches is the basic agreement that public health practice is a different field than clinical medicine with different motivating values, responsibilities, and goals.

One frequently noted difference between clinical medicine and public health is the obligation of public health to communities as well as individual patients. A clinician’s fiduciary duty to her patient requires a different lens than the public health professional’s duty to the community. Other important differences include the tools we use in our respective practices, the diversity of disciplines that contribute to public health, the broad range of responsibilities, and the number and variety of voices concerned with our decisions. These characteristics along with the very public—and often governmental—nature of public health contribute to a different set of values that constitute our moral foundation.

Ethics is the terrain of moral gray zones, where often there is no single clear and universally agreed-upon way forward. Skills in ethics and moral reasoning equip individuals to grapple with these gray zones and their inherent ambiguity. Public health is a practical endeavor, a solution-oriented field requiring action. It requires practical ethical judgments to drive solutions to ethical tensions. Practical ethical judgments require practical principles [2]. These practical principles rise from what we value as a society generally and as a profession specifically. They form what Beauchamp and Childress call our common morality and our professional morality, respectively [3]. Our common morality is the set of foundational norms, irrespective of ethical school or theory that all moral persons in a society judge as right standards for virtuous living. For example, despite widely divergent ideological foundations and differential understanding and valuation of “science” which underlie the current “pro” and “anti” perspectives about routine childhood immunization, both of these “camps” share an underlying moral commitment to protecting the health of their children.

DeGrazia expands the concept of common morality to include not simply a list of widely shared moral beliefs, rather a list of shared moral beliefs that are held up to ethical examination, questioned and refined through a process of reflective equilibrium [4]. This reflective examination—reflecting on our experiences and refining our moral judgments—enables us to reach a set of morally reasoned principles that guide the justification of our actions in a coherent way. This coherent justification is characterized by consistent, comprehensive, and cohesive judgments that are uninfluenced by conflicts of interest or conflicts of commitment.

To determine what foundational principles express our field’s professional morality, we must consider public health’s moral imperative [1]. What is our reason for being? What are our goals? How do we accomplish these goals? What public health is and what public health values will determine normative expectations about what we ought to do. From there we develop a common principled foundation to guide us in identifying and articulating the ethical dimensions of our work [1]. Once identified and articulated, we develop solutions for and take action on these ethical dimensions. The process for developing and implementing solutions is integral to the ethical practice of public health.

### Practice and content of modern public health

Modern public health commenced with the development of descriptive epidemiology by John Graunt in the 17^th^ century. It continued to develop as germ theory informed us about disease transmission and as public health policies began to gain favor in 19^th^ century England as a way to protect the health of entire communities at a time. As early physicians worked with sanitarians and engineers to devise ways to keep communities healthy, they relied on values reflected in the Hippocratic Oath to ground their work.

The World Health Organization (WHO) defines public health as, “all organized measures (whether public or private) to prevent disease, promote health, and prolong life among the population as a whole” [5]. This expansive definition places most things societies do—primary and secondary education, environmental protection, promotion of gender equality, and international development -- in the scope of public health. For the purpose of this discussion, we focus public health to include a set of activities more proximate to health, usually conducted under the auspices of national, state, and local governmental health agencies or aid organizations, that protect the health of, prevent harm to, and promote the development of the health of a community. These communal efforts address health needs that individuals cannot meet on their own. Public health accomplishes these efforts through practice, research, and policy.

#### Practice

A great deal of the work of public health happens through practice. Public health practice consists of numerous activities, most of which can be categorized into public health surveillance, recording of vital events, population-based health assessments, community-based prevention and health promotion programs, emergency preparedness and response, and evaluation of public health activities. Public health surveillance, recording of vital events and population-based health assessments (surveys and physical measurements) act as the eyes and ears of public health, providing important evidence for the level of well-being of communities.

Knowing what communicable and non-communicable conditions are affecting communities provides the foundation for public health action—the development and implementation of community-based prevention programs. Prevention efforts can be primary, which aim to prevent an infection, illness, or condition from occurring in the first place; they can be secondary, which aim to shorten the duration of a condition after infection or onset, often via screening for asymptomatic conditions; and they can be tertiary, which aim to prevent sequelae of an existing condition [6]. In recent years and in conjunction with the renewed emphasis on social determinants of health [7], primordial prevention has been defined as the activities designed to alter the systemic factors (such as social, economic, and environmental) that inhibit health [8].

Unlike the individual patient focus of the clinical encounter, public health prevention and intervention programs are community-based. These prevention programs are the backbone of much public health effort. Public health implements programs to prevent the onset or progression of a wide range of communicable and non-communicable conditions—from population-based newborn screening for metabolic disorders, to immunizations for communicable diseases across the lifespan, to fall-prevention and home care programs for older adults, to health promoting built environments, to toxic exposure mitigation.

Most recently, public health has actively engaged in emergency preparedness and response. In addition to concerns about emerging infectious diseases and bioterrorism, natural disasters such as fires, floods, and extreme weather can create health harms. To be best prepared to address health the aftermath of such emergencies, public health has joined other civic responders in preparedness planning and response.

As an evidence-based field, public health practice also includes monitoring and evaluation of activities to enable course correction and future planning. Evaluation data might come from surveillance, vital registration, population-based surveys, or other independent sources. The information gleaned from such evaluations is then fed back to public health program activities to improve effectiveness.

#### Research

Public health practitioners also conduct research that contributes to generalizable knowledge. Public health research, separate from program evaluation activities, includes clinical research, environmental research, behavioral research, intervention research, and prevention research. Some of the research we do involves examining outcomes for individuals; but much of it involves examining outcomes for communities. For example, at the start of the HIV epidemic in the 1980s, public health researchers examined what prevention interventions best reduced incidence of HIV infection among high risk populations. Numerous studies confirmed a decline in new infections among injection drug users in communities where public health provided clean and free injection equipment [9] and among persons at high risk where public health provided communications skill building and instruction on proper condom use [10]. Later, once pharmaceutical treatment was developed, public health researchers examined how the availability of HIV treatment affected the number of new infections among children born to infected mothers [11]. The outcomes in these studies included important community-level measures such as HIV incidence rates among specific subpopulations. Public health research demonstrated that a multi-disciplinary, complementary approach that includes both health promotion and biomedical aspects of disease control is effective in reducing the burden of a major public health challenge. In the case of HIV, these efforts must continue until a safe, effective, and inexpensive vaccine is developed. Results from public health research provide important information about the best way to focus both financial and human resources to combat a vast array of serious health threats to populations.

#### Policy

Policymaking is one of public health’s most essential and effective tools. Policies are requirements or restrictions that encumber individuals and communities to promote the public’s health. They can take the form of laws, regulations, agreements, rules, or procedures. Often policies are implemented nationally or at a smaller political subdivision such as a state or province. Depending on the nature of the public health activity or problem, some policies are implemented at an even smaller subdivision such as a city, town, or village. At the international level, efforts in the early 2000s led to WHO’s revision of the international health regulations, which went into effect in 2007, binding 196 nations to aid the international community in preventing, controlling, and responding to the international spread of disease [12]. Public health policies comprise a wide variety of topics including reportable conditions, immunization requirements, required prevention services, conditions warranting isolation and quarantine, and public safety measures related to the built environment.

Various health systems around the world incorporate public health activities and responsibilities differently, depending on the structure, financing, and availability of health care in a population. Where governments provide for the health care for all citizens, as is the case in most European countries, public health practice, research, and policy is often integrated as a component of the overall health system. Where governments do not provide for universal coverage for citizens, as is the case in most low- and middle-income countries as well as in the United States, public health activities are often conducted through distinct and separate systems. These systems can be governmental or non-governmental organizations.

### Public health in historical context: politics, power, science, and the state

A discussion of public health today, and of public health ethics, requires us to consider our historical roots [13]. This historical perspective foreshadows realities and tensions that might not be as obvious in contemporary public health, which takes for granted science and modern epidemiology. Infectious disease control at a population level long predates modern epidemiology and the germ theory of disease. It is historically intertwined with politics, power, and money-the practice of quarantine was developed in the 14th century as part of trade and political agreements among city states [14]. Yellow fever vaccine and the rise of the Pan-American Health Organization were linked to economic, political, and military action in Central America, including the construction of the Panama Canal [15,16].

The development of epidemiology itself required at least rudimentary statistics. This was intimately associated with the rise and consolidation of the modern state, where counting and controlling populations—including during epidemics—was at least as much about securing the legitimacy and authority of the state as about protecting the health of populations [17]. Modern scholars of public health such as Simon Szreter [18]. echo the views of the field’s founders, like Rudolph Virchow, that political action and protest by citizens and health workers were essential for the public benefits of public health [19].

Globally, public health is also fundamentally linked to colonialism. The notion of the “cordon sanitaire”, most recently demonstrated in attempts to contain the Ebola epidemic in western Africa [20], was an integral part of the French colonial presence in western Africa, [21] and similar concerns about the health of settler and local elite populations were echoed in disease control programs elsewhere [22,23]. The combination of terrifying epidemics and the visible, often military, presence of the state in trying to contain them has contributed to a range of popular fears and narratives and conspiracy theories of deliberate actions to harm specific communities and populations [24]. These narratives are particularly stark in relation to population control programs [25], and pandemics such as HIV [26].

Power, politics, and governance—especially the role of the state and relationships between state, citizens, and science—continue to influence public health practice and debates. Public health, like most benefits of modern society (including increases in life expectancy and wellbeing) would not be possible without a modern, ‘bureaucratic’ state to organize resources to provide for its population that which individuals cannot provide for themselves. Public health is necessarily linked to the state, to public policy, and ultimately to politics; this link raises issues and tensions that public health ethics must address. As demonstrated by a current debate, arguments against routine immunization reflect underlying libertarian perspectives that mistrust the state or at least strongly value strictly curtailing the power of the state. Opposing arguments reflect communitarian or utilitarian public health perspectives on balancing individual and collective goods-and reflect a generally more positive disposition to the role of the state and of science in informing public policy. Articulating the ethical basis for public health thus requires an appreciation of the common morality and professional morality underlying not only individual and population health, but also the relationships between citizens and the state. And this in turn entails a multidisciplinary approach that engages natural and health sciences as well as social, political, and human sciences.

### Public health’s professional morality

What, then, are the elements of professional morality underlying public health today? Public health is a practical and applied field. Our work is action- and solution-oriented. We value evidence-based results. We rely heavily on evidence to support just actions, many of which are aimed at righting inequities, especially with respect to health and related social capital. We value justice and equity. We enlist research ethics to ensure respectful treatment of both human research participants and non-human subjects. Public health is a participatory field requiring inputs from many agencies, individuals, and communities. We put a great deal of effort toward engaging with the communities we serve. We value transparency. Our service often is seated within a government or government-supported structure. The role of public policy in public health is crucial and requires a functioning governing process. We value and depend on public trust and collective action. Our ultimate goal is to reduce suffering and improve health to facilitate a full life for members of our communities.

In a review of 13 public health ethics frameworks published through 2010 several foundational values for the field of public health emerged, including an obligation to prevent harm and protect health, respect for individuals, least infringement, trust, transparency, confidentiality, production of benefits, justice, and equity [1]. These foundational values undergird the majority of public health ethics frameworks developed over the past 20 years, whether the framework was developed by public health practitioners out of a practical need or was based on a specific philosophical theory. That various frameworks are beginning to converge on a set of foundational values lends credibility to their applicability.

Several recent reflections on why public health exists and what it values have clarified several important facets of our professional morality: Health is necessary for human flourishing; there is a need to right inequities across health-promoting goods and services; and liberal governments are obligated to provide opportunities for individuals and communities in their population to access health.

A basic level of health is necessary for human flourishing so persons can seek a satisfying, autonomous life. To achieve this basic level of health, public health systems must exist to protect the health of individuals and communities, to prevent disease and injury, and to promote engagement of communities. Given that opportunities and resources for health are inequitably distributed, public health must seek to right this inequity. This inequity is the reason that the libertarian perspective that only negative rights—often referred to as the right to be left alone—fail many groups and individuals in nearly all societies. Righting inequity is based in a sense of social justice. Social justice arguments in public health have called for the provision of basic level of health for all persons [27]. The provision of such conditions through positive goals and negative constraints has been called the stewardship model by the U.K. Nuffield Council on Bioethics [28]. This model emerged from a comprehensive review of the roles and responsibilities of liberal governments in public health.

There is broad agreement that governments are stewards of their populations and are responsible for providing conditions that allow for its members to be healthy and productive. Given the role of policy and government in public health, the role of political philosophy likely has a substantial place as we seek a coherent system of ethical justification in our work [29]. Several authors have outlined or proposed the foundational role of solidarity [30-32] social justice [27], and communitarianism [33] in the professional morality of public health practice. The degree to which these values broaden our professional morality will depend on how communities interpret and value the role of public health.

Many of the ethical dimensions of public health are similar to the broader common morality seen in democratic or representative societies— such things as freedom, protection from harm, justice, and equal opportunity. In such societies, achieving this common morality requires cooperative schemes to improve efficiency and achievement of the population (e.g., education and transportation) or to care for citizens when they are unable to care for themselves (e.g., police protection, emergency response, and health care) [34]. There is a diversity of views—both between and within countries—with respect to how a government should be involved in protection, promoting solidarity, and equalizing opportunity. These views will affect how communities and populations prioritize the importance of these ethical foundations.

## Conclusion

Although some scholars have argued for a single unifying theoretical approach to support public health ethics, others have advocated for a more practical perspective. The aim here is not to align with one theoretical approach or another, rather, to consider the foundational values of public health practice order to identify the common moral governance of our work. Our profession’s morality—the set of norms shared by all public health professionals—is determined by what public health is and what we think it should be. As our aspirations for public health evolve, it is incumbent upon us to engage in reflective discourse to reach a new equilibrium about our moral foundation.
